# Vertebral Bone Marrow Heterogeneity Using Texture Analysis of Chemical Shift Encoding-Based MRI: Variations in Age, Sex, and Anatomical Location

**DOI:** 10.3389/fendo.2020.555931

**Published:** 2020-10-15

**Authors:** Michael Dieckmeyer, Daniela Junker, Stefan Ruschke, Muthu Rama Krishnan Mookiah, Karupppasamy Subburaj, Egon Burian, Nico Sollmann, Jan S. Kirschke, Dimitrios C. Karampinos, Thomas Baum

**Affiliations:** ^1^Department of Diagnostic and Interventional Neuroradiology, School of Medicine, Klinikum rechts der Isar, Technical University of Munich, Munich, Germany; ^2^Department of Diagnostic and Interventional Radiology, School of Medicine, Klinikum rechts der Isar, Technical University of Munich, Munich, Germany; ^3^Pillar of Engineering Product Development, Singapore University of Technology and Design, Singapore, Singapore; ^4^TUM-Neuroimaging Center, Klinikum rechts der Isar, Technical University of Munich, Munich, Germany

**Keywords:** bone marrow, magnetic resonance imaging, texture analysis, spine, proton density fat fraction

## Abstract

**Objective:** Vertebral bone marrow composition has been extensively studied in the past and shown potential as imaging biomarker for osteoporosis, hematopoietic, and metabolic disorders. However, beyond quantitative assessment of bone marrow fat, little is known about its heterogeneity. Therefore, we investigated bone marrow heterogeneity of the lumbar spine using texture analysis of chemical-shift-encoding (CSE-MRI) based proton density fat fraction (PDFF) maps and its association with age, sex, and anatomical location.

**Methods:** One hundred and fifty-six healthy subjects were scanned (age range: 20–29 years, 12/30 males/females; 30–39, 15/9; 40–49, 5/13; 50–59, 9/27; ≥60: 9/27). A sagittal 8-echo 3D spoiled-gradient-echo sequence at 3T was used for CSE-MRI-based water-fat separation at the lumbar spine. Manual segmentation of vertebral bodies L1-4 was performed. Mean PDFF and texture features (global: variance, skewness, kurtosis; second-order: energy, entropy, contrast, homogeneity, correlation, sum-average, variance, dissimilarity) were extracted at each vertebral level and compared between age groups, sex, and anatomical location.

**Results:** Mean PDFF significantly increased from L1 to L4 (35.89 ± 11.66 to 39.52 ± 11.18%, *p* = 0.017) and with age (females: 27.19 ± 6.01 to 49.34 ± 7.75%, *p* < 0.001; males: 31.97 ± 7.96 to 41.83 ± 7.03 %, *p* = 0.025), but showed no difference between females and males after adjustment for age and BMI (37.13 ± 11.63 vs. 37.17 ± 8.67%; *p* = 0.199). Bone marrow heterogeneity assessed by texture analysis, in contrast to PDFF, was significantly higher in females compared to males after adjustment for age and BMI (namely contrast and dissimilarity; *p* < 0.031), demonstrated age-dependent differences, in particular in females (*p* < 0.05), but showed no statistically significant dependence on vertebral location.

**Conclusion:** Vertebral bone marrow heterogeneity, assessed by texture analysis of PDFF maps, is primarily dependent on sex and age but not on anatomical location. Future studies are needed to investigate bone marrow heterogeneity with regard to aging and disease.

## Introduction

The non-mineralized bone compartment can be divided into red bone marrow which features higher vascularization and cellularity and is essential for hematopoiesis, and yellow bone marrow which has a higher fat content and important metabolic and endocrine functions (1, 2). The two bone marrow components can be quantitatively assessed using chemical shift encoding-based water–fat magnetic resonance imaging (CSE-MRI) and magnetic resonance spectroscopy (MRS). These two techniques allow for quantification of the proton density fat fraction (PDFF) while MRS additionally has been shown to enable the quantification of marrow fat composition (3–5).

Variations in bone marrow composition, assessed by quantitative MRI, have been demonstrated to be associated with a variety of diseases, such as osteoporosis, hematopoietic disorders, and metabolic disorders, such as obesity and type 2 diabetes mellitus (6–9). In osteoporosis, bone mineral density (BMD) is reduced and a negative correlation of BMD with bone marrow fat fraction as well as a positive correlation with bone marrow unsaturation has been established (10–14). Thus, MR-based quantification of vertebral bone marrow composition has been proposed as imaging biomarker for osteoporosis and fracture risk assessment (3, 14, 15). In addition, vertebral bone marrow composition has been recommended as an advanced surrogate marker for hematopoietic and metabolic disorders such as multiple myeloma and metabolic syndrome (16–20).

Previous studies showed that PDFF is a valid biomarker for fatty conversion of vertebral bone marrow and demonstrated a cranio-caudal increase in fat content for both adults and children (21, 22). An accelerated age-dependent fatty conversion of vertebral bone marrow was shown in females compared to males, particularly pronounced after menopause (23).

However, there are methods that allow quantitative assessment of bone marrow beyond PDFF and fatty acid unsaturation. Burian et al. showed that CSE-MRI can be used for the assessment of vertebral bone marrow heterogeneity using texture analysis (24).

The texture of an image or three-dimensional imaging dataset is characterized by the spatial arrangement of voxels with different intensities. Texture analysis intends to quantify features reflecting repetitive patterns in the image as a function of the spatial gray-level variation. First-order, or global, features provide information related to the gray-level distribution of the image without carrying any information about the relative positions of the gray-levels within the image. This information is obtained from second-order features which can be extracted from the gray-level co-occurrence matrix (GLCM) as described by Haralick et al. (25). Texture features have previously been applied in different biomedical imaging modalities, e.g., for computed tomography (CT)-based analysis of bone microstructure, CT-based fracture risk assessment, and diagnostic support in mammography (26–28), in multi-modal oncologic imaging analysis (29–31) as well as for MRI-based computer-aided diagnosis and classification of vertebral compression fractures (32, 33). In a recent preliminary study, CSE-MRI-based texture analysis demonstrated an increased vertebral bone marrow heterogeneity after menopause and the second-order features contrast and dissimilarity were shown to differentiate post- from premenopausal equally well as PDFF (24). However, this previous study only included a relatively small number of female subjects and vertebral bone marrow heterogeneity has not been characterized in greater detail.

Therefore, the purpose of our study was to further investigate the heterogeneity of the lumbar vertebral bone marrow with regard to anatomical location, sex, and age in a larger cohort using texture analysis of CSE-MRI-based PDFF maps.

## Materials and Methods

### Subjects

Written informed consent was obtained from all subjects before participation in the study which was approved by the local institutional committee for human research. Healthy subjects older than 20 years of age were included in this study as outlined previously (23). The following exclusion criteria were applied: history of pathological bone changes such as hematological or metabolic bone disorders other than osteoporosis, history of diabetes, and contraindications for MRI. In total, 156 healthy subjects were recruited: age range 20–29 years (age group 1), 12/30 males/females; 30–39 years (age group 2), 15/9 males/females; 40–49 years (age group 3), 5/13 males/females; 50–59 years (age group 4), 9/27 males/females; 60 years and older (age group 5): 9/27 males/females.

### MR Imaging

All subjects were scanned using the same 3T MRI system (Ingenia, Philips Healthcare, Best, The Netherlands). For chemical shift-encoding based water–fat separation at the lumbar spine, a sagittal eight-echo 3D spoiled gradient echo sequence was performed using the built-in-the-table posterior coil elements (12-channel array). The eight echoes of this sequence are acquired in a single repetition time (TR) using non-flyback (bipolar) read-out gradients and the following imaging parameters: TR/TE1/ΔTE = 11/1.4/1.1 ms, field of view (FOV) = 220 × 220 × 80 mm, acquisition matrix size = 224 × 224 × 20, acquisition voxel size = 0.98 mm × 0.98 mm × 4.00 mm, receiver bandwidth = 1,527 Hz/pixel, frequency direction = anterior-posterior (to minimize breathing artifacts), 1 average, scan time = 1 min and 17 s. A flip angle of 3° was used to minimize T1-bias effects (34, 35).

### Vertebral Bone Marrow Fat Quantification

**Figure 1:**
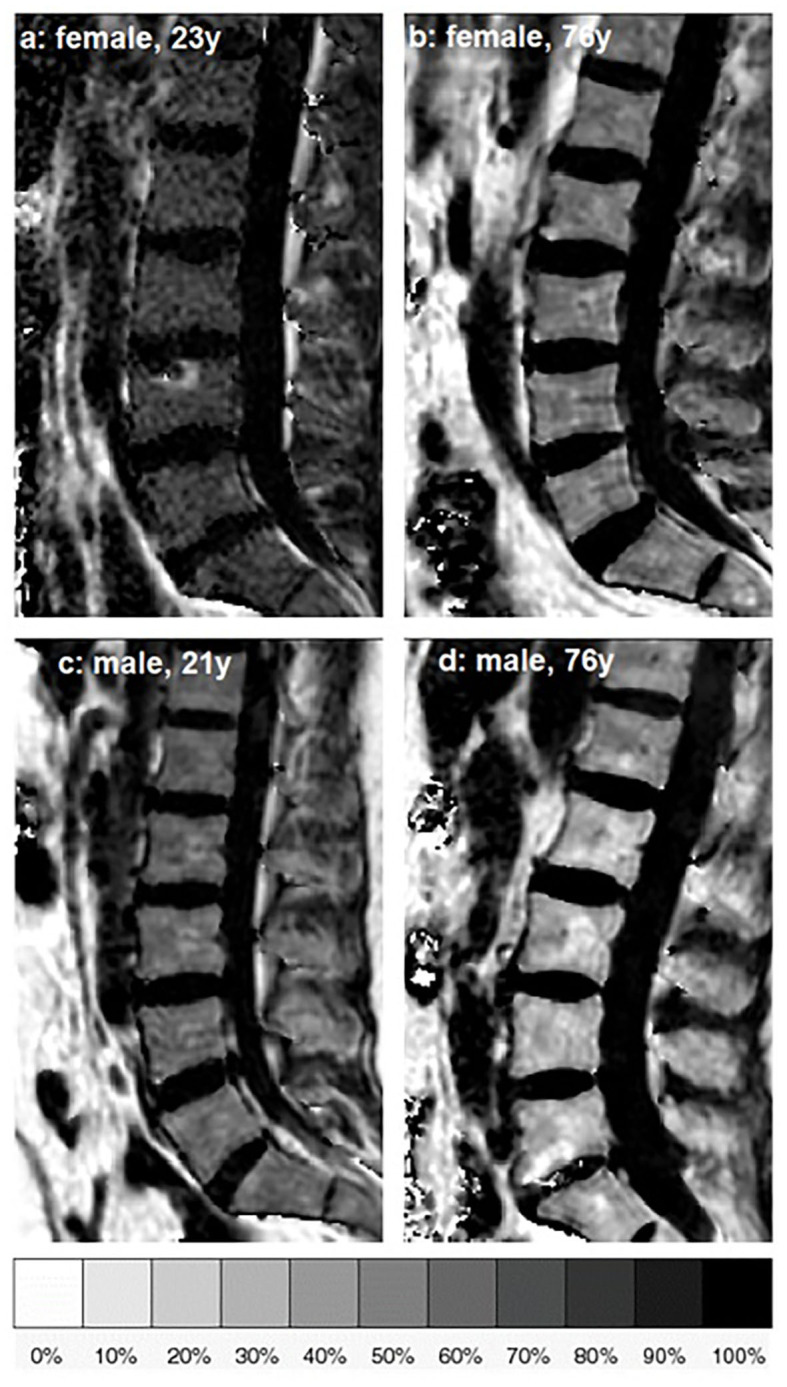
Representative sagittal proton density fat fraction (PDFF) [%] maps of one female and one male subject of age group 1 **(a,c)** and age group 5 **(b,d)**, respectively.

**Figure 2:**
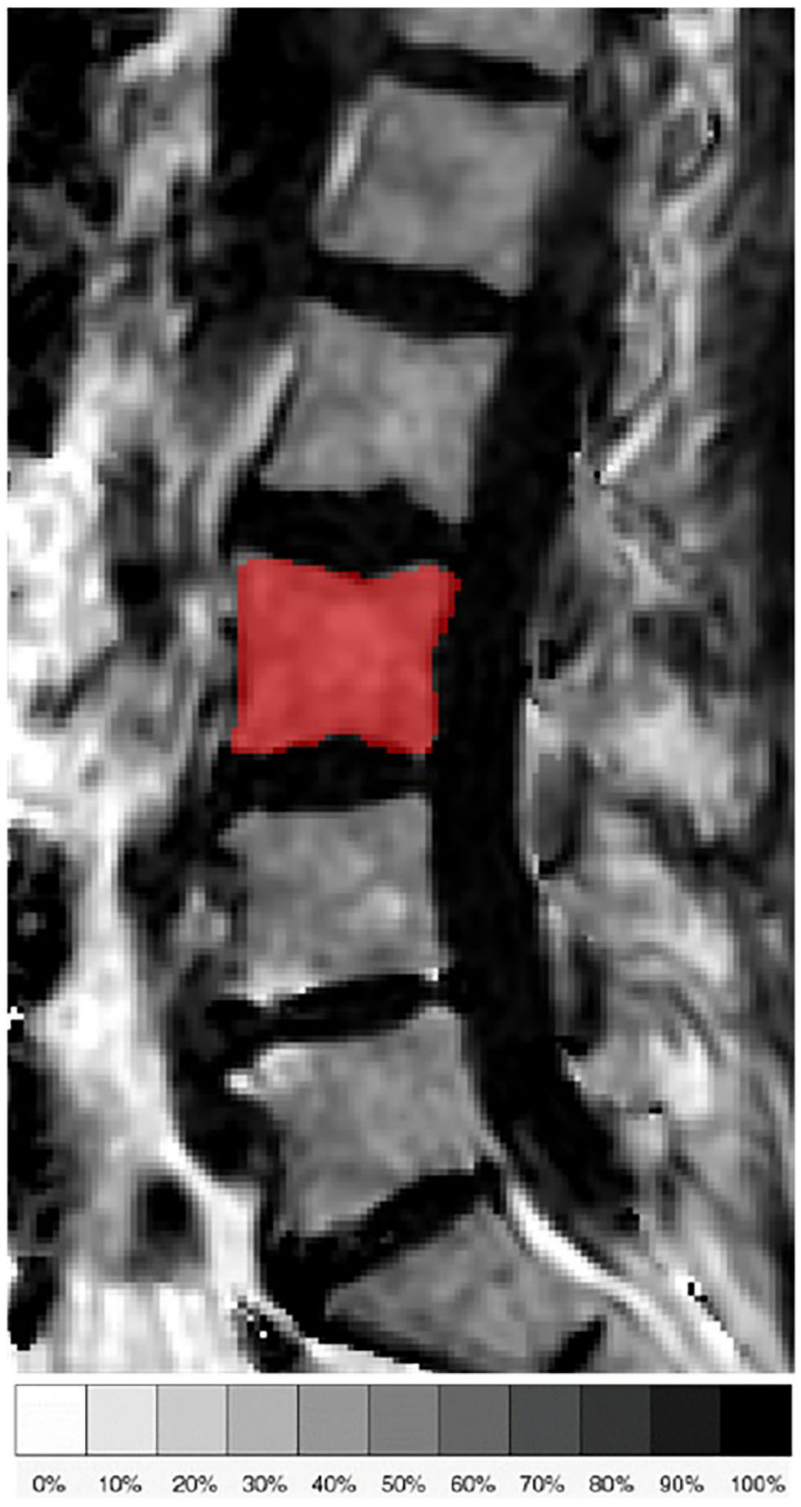
Representative sagittal slice of the segmentation of the L3 vertebral body in the proton density fat fraction (PDFF) map of a 71-year old female subject.

The fat quantification routine of the MRI system vendor was used for on-line processing of the gradient echo imaging data including the following steps: first, phase error correction; second, complex-based water–fat decomposition using a precalibrated seven-peak fat spectrum and a single T2^*^ to model the signal variation with echo time (36, 37). The imaging-based PDFF map was computed as the ratio of the fat signal over the sum of fat and water signals. Figure 1 shows representative PDFF maps. The vertebral bodies L1 to L4 were included in the analysis and manually segmented by a radiologist with 3 years of experience in spine imaging (Figure 2). The segmentations did not include Schmorl nodes or cortical bone but comprised the basivertebral veins which could be considered as a potential source of quantification bias. However, the segmentations are consistent with previous publications (21, 23). In order to avoid the inclusion of degenerative alterations, such as Modic changes, endplate-associated sclerotic changes were excluded. Segmentation was performed on the PDFF maps by using the free open-source software Medical Imaging Interaction Toolkit (MITK), developed by the Division of Medical and Biological Informatics, German Cancer Research Center, Heidelberg, Germany (www.mitk.org). PDFF values were calculated individually for each segmented vertebral level as well as for the entire segmentation from L1 to L4 to obtain average values for each subject.

### Texture Analysis

Texture analysis was performed on the PDFF maps of the segmented vertebral bodies. Three global features (variance, skewness, kurtosis) and the following eight second-order features were extracted: Energy, entropy, contrast, homogeneity, and correlation were calculated according to (25), variance and sum-average according to (38) and dissimilarity according to (39).

Global features were extracted from intensity histograms. The ideal number of bins used in the histogram analysis was calculated by a combination of different methods (40–42).

Second-order features were extracted using gray-level GLCM analysis (25). As a preprocessing step, gray level quantization of the PDFF maps was performed to prevent sparseness by normalizing the image intensities using 100 equally sized bins and the minimum and maximum gray levels present, corresponding to values of 0 and 100%, respectively.

GLCM is obtained by computing the joint probability of two adjacent voxel intensities at a given offset *d* = (*dx, dy, dz*) and angular directions θ = (0, 45, 90, and 135°). *dx, dy*, and *dz* denote the displacement along the *x*-, *y*-, and *z*-axis, respectively.

For 3D-GLCM analysis, the co-occurrence probabilities of voxel intensities were computed from the 26 neighbors, aligned in 13 directions taking into account discretization length differences. The mean value of the features computed from the 13 directions ensures the rotation invariance. Image preprocessing, including isotropic resampling, gray level uniform quantization and texture analysis were performed using MATLAB 2017 (MathWorks Inc., Natick, MA, USA) and a radiomics toolbox (https://github.com/mvallieres/radiomics/) (29–31).

### Statistical Analysis

The statistical analyses were performed with SPSS (SPSS Inc., Chicago, IL, USA). All tests were done using a two-sided 0.05 level of significance.

The Kolmogorov-Smirnov test indicated normally distributed data for the majority of parameters.

Mean and standard deviation (SD) of PDFF and texture features were calculated for L1 to L4 and a one-way analysis of variance (ANOVA) was performed to test for any significant differences between the four vertebral levels. Bonferroni-adjusted *post-hoc* analysis was used for pairwise comparisons between the four vertebral levels.

Furthermore, mean and SD of PDFF and texture features, averaged from L1 to L4, were calculated and compared with respect to sex, using a general linear model to adjust for age and body mass index (BMI).

Lastly, mean and SD of PDFF and texture features, averaged from L1 to L4, were calculated for the five age groups and a one-way ANOVA was performed to test for any significant differences between the age groups. This was done separately for female and male subjects. Bonferroni-adjusted *post-hoc* analysis was used for pairwise comparisons between the five age groups.

## Results

### Study Population

The study population consisted of 100 female subjects with an age of 46.6 ± 17.1 years and 56 male subjects with an age of 42.9 ± 15.5 years. The age difference between females and males was not significant (*p* > 0.05). BMI was significantly (*p* < 0.05) different between females and males (Table 1).

**Table 1:** Mean and standard deviation (SD) of subject characteristics, proton density fat fraction (PDFF), and analyzed texture parameters, averaged from L1 to L4 and grouped by sex (0: male, *n* = 50; 1: female, *n* = 106).

	**Sex**	**Mean**	**SD**	***p*-value**
Age	Male	42.90	15.48	0.198
	Female	46.59	17.15	
BMI	Male	28.25	4.62	0.016^*^
	Female	26.09	5.20	
PDFF	Male	37.17	8.67	0.199
	Female	37.13	11.63	
Variance (global)	Male	87.81	12.32	0.001^*^
	Female	74.57	10.49	
Skewness (global)	Male	−0.3846	0.5505	0.004^*^
	Female	−0.1829	0.5686	
Kurtosis (global)	Male	1.171	0.810	0.425
	Female	0.933	0.750	
Energy [10^−3^]	Male	1.53	0.45	0.212
	Female	1.40	0.44	
Contrast	Male	49.86	10.01	0.031^*^
	Female	52.05	9.70	
Entropy	Male	10.20	0.35	0.088
	Female	10.30	0.34	
Homogeneity	Male	0.290	0.022	0.043^*^
	Female	0.289	0.021	
Correlation	Male	0.823	0.047	0.195
	Female	0.828	0.046	
SumAverage [10^−3^]	Male	4.92	0.57	0.295
	Female	4.88	0.73	
Variance [10^−3^]	Male	15.10	5.10	0.993
	Female	16.10	4.80	
Dissimilarity	Male	5.097	0.577	0.018^*^
	Female	5.229	0.547	

Differences of PDFF and texture parameters between male and female subjects, adjusted for age and BMI, were compared using a general linear model (

* *indicates p < 0.05)*.

### PDFF Measurements

**Figure 3:**
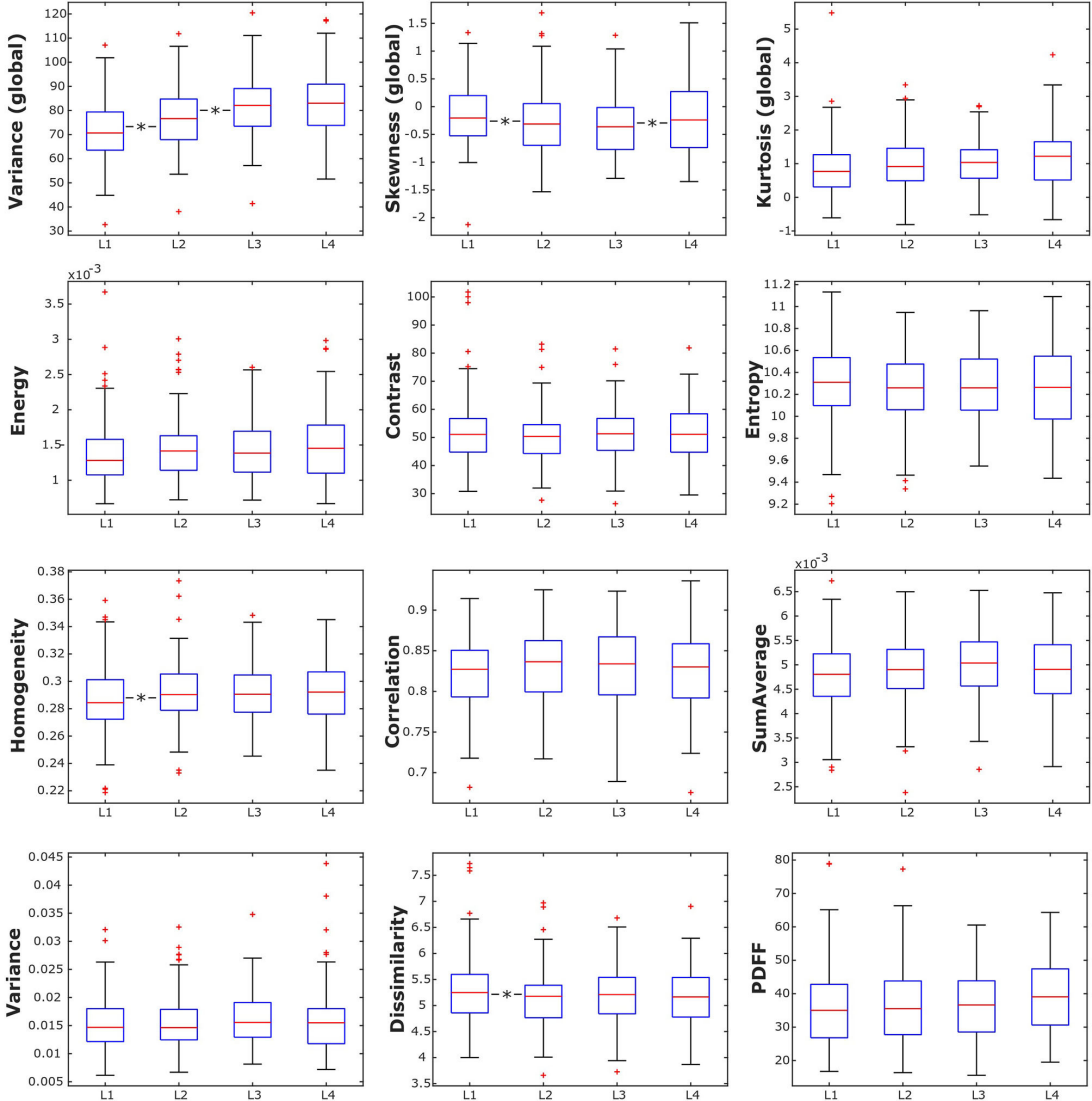
Box plots of the analyzed texture parameters and proton density fat fraction (PDFF) of all subjects, grouped by lumbar vertebral body. ^*^indicates stat. significant difference (*p* < 0.05) between consecutive lumbar vertebrae.

Mean PDFF for all subjects combined showed a statistically significant difference depending on vertebral location (*p* < 0.05), increasing from 35.89 ± 11.66% in L1 to 39.52 ± 11.18% in L4 (Table 2, Figure 3). Significant differences between consecutive vertebral levels are indicated with ^*^ in Figure 3.

**Table 2:** Mean and standard deviation (SD), and number of samples (*N*) of the analyzed texture parameters and proton density fat fraction (PDFF), grouped by vertebral location; *p*-values for test of differences between vertebral locations using analysis of variance [ANOVA; ^*^indicates stat. significant difference (*p* < 0.05) between any of the vertebral locations].

	**L1 (*****N*** **=** **145)**		**L2 (*****N*** **=** **153)**		**L3 (*****N*** **=** **155)**		**L4 (*****N*** **=** **156)**		***p*-value**
	**Mean**	**SD**	**Mean**	**SD**	**Mean**	**SD**	**Mean**	**SD**	**(ANOVA)**
Variance (global)	72.02	11.97	77.21	12.09	81.98	11.74	83.46	11.89	<0.001^*^
Skewness (global)	−0.1538	0.5339	−0.2845	0.5574	−0.3598	0.5182	−0.1853	0.6429	0.006^*^
Kurtosis (global)	0.8491	0.7743	0.9782	0.7637	1.0238	0.6674	1.1738	0.8628	0.004^*^
Energy [10^−3^]	1.370	0.460	1.440	0.430	1.430	0.400	1.512	0.490	0.065
Contrast	52.17	12.16	50	8.62	51.53	9.07	51.74	9.26	0.239
Entropy	10.29	0.36	10.25	0.32	10.29	0.31	10.25	0.37	0.620
Homogeneity	0.2863	0.0239	0.2919	0.0209	0.2908	0.0198	0.2923	0.0206	0.060
Correlation	0.8211	0.0454	0.831	0.0436	0.8301	0.0491	0.8236	0.0483	0.186
SumAverage [10^−3^]	4.81	0.75	4.88	0.66	5.00	0.63	4.90	0.71	0.106
Variance [10^−3^]	15.34	4.60	15.7	4.62	16.19	4.62	15.91	5.76	0.509
Dissimilarity	5.269	0.666	5.127	0.515	5.182	0.516	5.174	0.531	0.174
PDFF [%]	35.89	11.66	36.09	11.02	37.15	10.72	39.52	11.18	0.017

PDFF, averaged from L1 to L4, showed no statistically significant difference between female and male subjects (37.13 ± 11.63 vs. 37.17 ± 8.67%; Table 1).

**Figure 4:**
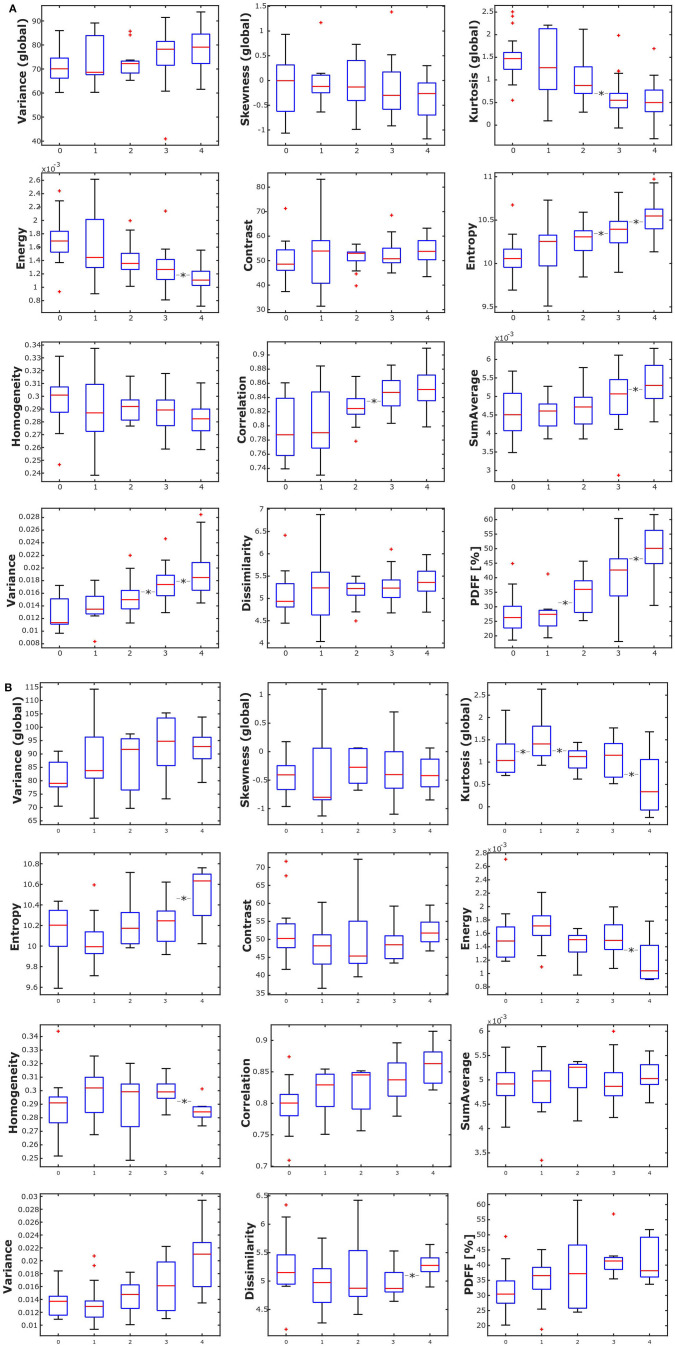
**(A)** Box plots of the analyzed texture parameters and proton density fat fraction (PDFF) of female subjects, grouped by age group (1: age 20–29 years, 2: age < 30–39 years, 3: 40–49 years, 4: age 50–59 years, 5: age ≥ 60 years). ^*^Indicates stat. significant difference (*p* < 0.05) between consecutive age groups. **(B)** Box plots of the analyzed texture parameters and proton density fat fraction (PDFF) of male subjects, grouped by age group (1: age 20–29 years, 2: age < 30–39 years, 3: 40–49 years, 4: age 50–59 years, 5: age ≥ 60 years). ^*^Indicates stat. significant difference (*p* < 0.05) between consecutive age groups.

In female subjects, PDFF, averaged from L1 to L4, showed a statistically significant difference depending on age group (*p* < 0.05), increasing from 27.19 ± 6.01% in age group 1 (20–29 years) to 49.34 ± 7.75% in age group 5 (≥ 60 years) (Table 3A, Figure 4A). In male subjects, PDFF, averaged from L1 to L4, showed a statistically significant difference depending on age group (*p* < 0.05), increasing from 31.97 ± 7.96% in age group 1 (20–29 years) to 42.04 ± 6.06 % in age group 4 (50–59 years) and plateauing at 41.83 ± 7.03% in age group 5 (≥ 60 years) (Table 3A, Figure 4A). Significant differences between consecutive age groups are indicated with ^*^ in Figures 4A,B.

**Table 3A:** **(Females)** Mean, standard deviation (SD), and number of samples (*N*) of texture parameters and proton PDFF of female subjects, grouped by age (1: age 20–29 years, 2: age < 30–39 years, 3: 40–49 years, 4: age 50–59 years, 5: age ≥ 60 years); *p*-values for test of differences between age groups using analysis of variance (ANOVA; ^*^indicates stat. significant difference between any of the age groups).

**Age group**	**1 (*****N*** **=** **30)**		**2 (*****N*** **=** **9)**		**3 (*****N*** **=** **13)**		**4 (*****N*** **=** **27)**		**5 (*****N*** **=** **27)**		***p*-value**
	**Mean**	**SD**	**Mean**	**SD**	**Mean**	**SD**	**Mean**	**SD**	**Mean**	**SD**	**(ANOVA)**
Variance (global)	71.33	7.31	74.71	10.31	72.78	6.05	75.2	10.33	78.4	8.32	<0.001^*^
Skewness (global)	−0.1083	0.5618	−0.0222	0.5204	−0.0442	0.4933	−0.1866	0.5247	−0.3678	0.395	<0.001^*^
Kurtosis (global)	1.4753	0.4368	1.3035	0.7604	1.0155	0.5476	0.6237	0.4265	0.5238	0.4064	<0.001^*^
Energy [10^−3^]	1.704	0.298	1.625	0.554	1.429	0.270	1.271	0.275	1.136	0.209	<0.001^*^
Contrast	49.74	6.53	52.47	15.05	51.13	4.84	52.14	5.15	54.25	5.04	0.016^*^
Entropy	10.06	0.19	10.16	0.36	10.26	0.19	10.4	0.2	10.52	0.19	<0.001^*^
Homogeneity	0.296	0.0173	0.2913	0.0315	0.2923	0.0123	0.2878	0.0144	0.2811	0.0117	<0.001^*^
Correlation	0.7949	0.0405	0.8047	0.053	0.8265	0.0247	0.8462	0.0221	0.8542	0.0264	<0.001^*^
SumAverage [10^−3^]	4.555	0.607	4.552	0.461	4.674	0.610	4.93	0.648	5.385	0.556	<0.001^*^
Variance [10^−3^]	12.58	2.37	13.83	2.69	15.33	3.05	17.49	2.55	19.05	3.84	<0.001^*^
Dissimilarity	5.062	0.415	5.222	0.849	5.154	0.292	5.248	0.322	5.397	0.300	<0.001^*^
PDFF [%]	27.19	6.01	27.27	6.19	34.55	6.83	40.51	9.73	49.34	7.75	<0.001^*^

### Texture Analysis

Combining all subjects, the global texture features variance, skewness, and kurtosis showed statistically significant anatomical variations from L1 to L4 (*p* < 0.05). However, only variance and kurtosis showed a consistent increasing trend from L1 to L4. None of the second-order texture features showed a significant anatomical variation from L1 to L4 (*p* > 0.05) (Table 2, Figure 3). Significant differences between consecutive vertebral levels are indicated with ^*^ in Figures 4A,B.

Averaged from L1 to L4 and adjusted for age and BMI, the global features variance and skewness as well as the second-order features contrast, dissimilarity and homogeneity showed statistically significant differences between female and male subjects (Table 1).

In female subjects, all texture features, averaged from L1 to L4, showed statistically significant differences between age groups (*p* < 0.05). A consistent trend was shown for the global feature kurtosis (decreasing with age) and the second-order features energy (decreasing with age), entropy (increasing with age), correlation (increasing with age), and variance (increasing with age) (Table 3A, Figure 4A). In male subjects, all texture features, averaged from L1 to L4, except for the global feature skewness and the second-order feature variance showed statistically significant differences between age groups (*p* < 0.05). However, a consistent trend was shown only for the second-order feature correlation (increasing with age) (Table 3B, Figure 4B). Significant differences between consecutive age groups are indicated with ^*^ in Figures 4A,B.

**Table 3B:** **(Males)** Mean, standard deviation (SD), and number of samples (*N*) of texture parameters and proton PDFF of male subjects, grouped by age (1: age 20–29 years, 2: age < 30–39 years, 3: 40–49 years, 4: age 50–59 years, 5: age ≥ 60 years); *p*-values for test of differences between age groups using analysis of variance (ANOVA; ^*^indicates stat. significant difference between any of the age groups).

**Age group**	**1 (*****N*** **=** **12)**		**2 (*****N*** **=** **15)**		**3 (*****N*** **=** **5)**		**4 (*****N*** **=** **9)**		**5 (*****N*** **=** **9)**		***p*-value**
	**Mean**	**SD**	**Mean**	**SD**	**Mean**	**SD**	**Mean**	**SD**	**Mean**	**SD**	**(ANOVA)**
Variance (global)	81.04	6.96	87.5	12.82	86.56	11.87	93.65	11.15	91.92	7.25	<0.001^*^
Skewness (global)	−0.4286	0.3256	−0.4515	0.6382	−0.2697	0.3315	−0.2953	0.5752	−0.3947	0.3213	0.610
Kurtosis (global)	1.1811	0.4819	1.5958	0.5211	1.0651	0.3052	1.1229	0.459	0.4707	0.6992	<0.001^*^
Energy [10^−3^]	1.560	0.428	1.712	0.291	1.425	0.265	1.533	0.298	1.168	0.308	<0.001^*^
Contrast	52.56	8.87	47.4	6.5	50.22	12.8	48.53	5.01	52.25	4.08	0.041^*^
Entropy	10.15	0.25	10.05	0.24	10.22	0.29	10.22	0.24	10.51	0.26	<0.001^*^
Homogeneity	0.2881	0.0234	0.3	0.0172	0.2899	0.0268	0.2998	0.01	0.2848	0.008	0.003^*^
Correlation	0.7975	0.0426	0.816	0.0348	0.8206	0.0411	0.8385	0.0363	0.8589	0.0314	<0.001^*^
SumAverage [10^−3^]	4.879	0.436	4.844	0.552	5.032	0.502	4.973	0.558	5.074	0.338	0.372
Variance [10^−3^]	13.51	2.24	13.49	3.20	14.42	2.99	16.01	4.21	19.96	5.00	<0.001^*^
Dissimilarity	5.247	0.574	4.937	0.394	5.157	0.765	4.982	0.27	5.289	0.221	0.016^*^
PDFF [%]	31.97	7.96	35.28	6.67	38.2	14.87	42.04	6.06	41.83	7.03	0.025^*^

## Discussion

In the present study, we observed that vertebral bone marrow heterogeneity, assessed by texture analysis of PDFF maps, exhibits sex-specific differences. Significant differences between age groups were found in a considerable subset of the analyzed texture features, particularly in women. In contrast to PDFF, bone marrow heterogeneity shows no gradient along the lumbar spine.

In 1973, Haralick et al. (25) described the computation of 14 textural features containing information about characteristics, such as homogeneity, contrast, linear structure, or complexity, on photo-micrographs and satellite images and applied them for image-classification. Since then, texture features have been increasingly used based on a variety of medical imaging modalities, with a strong focus on oncologic applications (27, 29–31, 43–45). Shape, texture and statistical features of T1-weighted MR images of the lumbar spine have also been shown to improve diagnostic accuracy and classification results of vertebral compression fractures (32, 33). Furthermore, texture analysis has been reported as a reproducible tool for the quantitative assessment of fatty infiltration of paraspinal muscles in patients suffering from lumbar spinal stenosis based on axial T2-weighted MR images (46). In this study, we performed texture analysis of vertebral bone marrow on CSE-MRI-based PDFF maps, an approach which has been shown to be feasible by Burian et al. (24). We took a texture feature processing tool which used positron emission tomography (PET) and non-quantitative MR images of the extremities as well as the head and neck region in the context of radiomics models and tailored it to be applied to PDFF maps of vertebral bone marrow which, by themselves are already quantitative. The voxels of the PDFF maps assume gray-level values corresponding to a range between 0 and 100%. We normalized all image intensities to this same range using the same gray-level quantization for each dataset. Furthermore, the same MRI protocol and scan parameters and resolution were used for each subject ensuring comparable signal-to-noise ratios throughout the scans. As a result, these measures ensure good comparability of the extracted features among the, compared to previous studies, relatively large number of subjects (24, 47).

In this larger-scale study, we found that bone marrow heterogeneity, assessed by the second-order textural features contrast and dissimilarity, is significantly higher in females compared to males, while PDFF itself showed no significant difference between females and males averaged over all age groups. We also found a sex-dependent statistical significant difference in the second-order textural feature homogeneity. However, it was only marginal and can be considered negligible. One potential pathophysiologic explanation for the revealed sex-dependent difference may involve the conversion process from red to yellow marrow. According to Scheller et al. vertebral bone marrow adipose tissue is highly regulated and actively involved in hematopoiesis and skeletal remodeling (1). Several human and animal studies found sex-dependent differences regarding the relationship between marrow fat and bone which were potentially attributed to differences in sex hormone levels (6, 13, 48–50). So it stands to reason that the revealed sex-dependent differences in bone marrow heterogeneity may also be due to the effects of higher estrogen and testosterone levels in males compared to females, particularly in older subjects. Our results could suggest that, beyond the known sex-dependent differences in bone marrow fat content, there are also differences in the conversion process from red to yellow marrow. One potential confounder could be degenerative alterations of the vertebral bodies. Females are known to have a higher prevalence of Modic changes than males which could result in higher heterogeneity measurements (51). However, more severe visible Modic changes were excluded in the segmentations of the vertebral bodies, minimizing this potentially confounding effect.

In addition, our results showed an age dependence of some textural features suggesting an increase in vertebral bone marrow heterogeneity with age. While textural features of bone marrow have not been investigated with regard to age before, heterogeneity shows a similar behavior to PDFF increasing with age in our study, confirming previous results in children and adults (22, 23).

Previous studies have also demonstrated an increase in PDFF from cervical to lumbar vertebral bodies showing that conversion from red to yellow marrow is occurring first at lower vertebral levels (21–23). This could be confirmed in our study. However, in contrast to increasing PDFF from L1-4, textural features did not show a spatial gradient further supporting the hypothesis of Burian et al. of an anatomically homogeneous fatty conversion of bone marrow along the spine (24).

To the best of our knowledge, the present work is the first study investigating the association of bone marrow heterogeneity with sex and age. We found an association of bone marrow heterogeneity with sex and age, admittedly with varying degrees of statistical significance. The clinical relevance of these findings is still unclear. Therefore, an important and logical next step is to investigate these connections in subjects suffering from musculoskeletal and metabolic diseases affecting bone marrow structure and composition, such as osteoporosis and type 2 diabetes (3, 6–9, 15).

Our study is not without limitations. First, the number of female subjects was considerably higher than the number of male subjects, and some of the age groups comprised only a relatively small number of subjects. To more reliably characterize the revealed sex- and age-dependent differences in bone marrow textural features, the inclusion of additional subjects resulting in a more balanced sex and age distribution would be desirable. Second, the present study has a cross-sectional design with the inherent limitations. However, it could be helpful in designing a future longitudinal study, in particular for the characterization of changes in bone marrow heterogeneity related to age and diseases of high clinical interest.

## Conclusion

In the present study, we found that texture features of vertebral bone marrow, assessed by analysis of CSE-MRI-based PDFF maps of the lumbar spine, exhibit sex-, and age-specific differences. In contrast to PDFF, bone marrow shows no significant anatomical variation regarding textural features along the lumbar spine. Hence, vertebral bone marrow heterogeneity seems to be primarily sex- and age-dependent. Further studies are needed to investigate bone marrow heterogeneity during aging and disease progression in order to investigate whether texture analysis of bone marrow can improve the clinical impact of quantitative MRI of the spine.

## Data Availability Statement

The data supporting the conclusions of this article will be made available by the authors at reasonable request.

## Ethics Statement

The studies involving human participants were reviewed and approved by Ethikkommision der Technischen Universität München. The participants provided their written informed consent to participate in this study.

## Author Contributions

MD: concept of study design, subject recruitment, data acquisition, data post-processing, statistical analysis, and drafting the manuscript. DJ: subject recruitment, data acquisition, and critical revision of manuscript. SR: data acquisition and critical revision of manuscript. MM and KS: data post-processing and critical revision of manuscript. EB: subject recruitment and critical revision of manuscript. NS: critical revision of manuscript. JK: critical revision of manuscript and project supervision. DK: subject recruitment, data post-processing, and critical revision of manuscript. TB: concept of study design, subject recruitment, data post-processing, statistical analysis, drafting the manuscript, and leading role in critical revision of manuscript.

## Conflict of Interest

The authors declare that the research was conducted in the absence of any commercial or financial relationships that could be construed as a potential conflict of interest.

## Abbreviations

ANOVA: analysis of variance
BMD: bone mineral density
BMI: body mass index
CT: computed tomography
FOV: field of view
GLCM: gray-level co-occurrence matrix
L1: first lumbar vertebra
L4: fourth lumbar vertebra
MITK: Medical Imaging Interaction Toolkit
MRI: magnetic resonance imaging
MRS: magnetic resonance spectroscopy
PDFF: proton density fat fraction
PET: positron emission tomography
SD: standard deviation
T1: longitudinal relaxation time
T2: transverse relaxation time
T2^*^: effective transverse relaxation time
TE: echo time
ΔTE: echo time step
TR: repetition time.
